# Construction of a risk model based on m5C-associated lncRNAs to predict the prognosis in renal cell carcinoma

**DOI:** 10.1097/MD.0000000000043052

**Published:** 2025-07-04

**Authors:** Baosai Lu, Jin Wu, Yalin Niu, Yuewei Yin, Chenming Zhao

**Affiliations:** a Department of Urology, The Second Hospital of Hebei Medical University, Shijiazhuang, China.

**Keywords:** 5-methylcytosine, biomarker, long-noncoding RNAs, prognosis, renal cell carcinoma

## Abstract

Renal cell carcinoma (RCC) is one of the most common tumors of the urinary system, and its outcomes vary widely among individuals, primarily due to different molecular characteristics. Both 5-methylcytosine (m5C) methylation and long noncoding RNAs (lncRNAs) play crucial roles in the epigenetics of RCC and may serve as biomarkers for predicting prognosis. Clinical information and transcriptome data of patients with RCC were extracted from the The Cancer Genome Atlas database. Using least absolute shrinkage and selection operator analysis and multivariate Cox regression, m5C-related lncRNAs were filtered. We then built a prognostic prediction model based on m5C-related lncRNAs. The model was analyzed for its predictive role in overall survival (OS) and response to targeted and immunotherapeutic treatments. We selected 3 lncRNAs, HM13-IT1, COLCA1, and AC010285.3, to construct a predictive model that categorizes patients into high-risk and low-risk groups. The results indicated that the high-risk group exhibited a significantly poorer OS than the low-risk group, and upon validation, it was identified as an independent risk factor. Through gene ontology enrichment analysis, this model was found to be closely associated with tumor immune function. The high-risk group showed higher tumor mutation burden and tumor immune dysfunction and exclusion scores, suggesting poorer response to immunotherapy. Additionally, the high-risk group exhibited reduced responsiveness to sorafenib. The predictive model for RCC can accurately forecast the prognosis of RCC, offering new tools for personalized diagnosis and treatment of individual patients.

## 1. Introduction

Renal cell carcinoma (RCC) is one of the most common malignant diseases, accounting for approximately 3% of all malignant tumors, with increasing incidence.^[1,2]^ RCC has become the ninth most common cancer in men and the fourteenth in women. Based on pathology, it is mainly divided into clear cell carcinoma (70%), papillary carcinoma (10%–15%), chromophobe carcinoma (5%), and other rare types.^[3]^ Despite technological advances leading to a decrease in the mortality rate of kidney cancer in recent years, in 2020, there were still 179,368 patients worldwide who succumbed to kidney cancer. Because early stage kidney cancer usually presents with no obvious symptoms, when discovered, RCC is already at an advanced stage. Moreover, advanced kidney cancer currently lacks effective treatment options that are insensitive to radiotherapy and chemotherapy. This poses a severe threat to people’s lives and imposes a significant economic burden.^[4]^ Therefore, early diagnosis is important for improving the prognosis of RCC, and the exploration of new predictive biomarkers and novel treatment targets is warranted.

Recent research has revealed that RNA 5-methylcytosine (m5C) may play a crucial role in the occurrence and development of various tumors.^[5]^ M5C modification influences the expression of various genes by regulating the stability and translation of multiple RNA types, including mRNA, rRNA, tRNA, and ncRNA, thereby altering the biological characteristics and functions of cells. This kind of RNA modification process is reversible and is primarily governed by the m5C methyltransferase complex (writers), including the NSUN family (NSUN1-NSUN7) and TRDMT1 (DNMT2), m5C demethylase enzymes (erasers) such as ALKBH1, and the TET family and m5C reading proteins (readers) such as ALYREF and YBX1.^[6]^ Several studies have found that m5C-regulated proteins can participate in immune infiltration of renal cancer cells and are associated with patient prognosis.^[7,8]^

In humans, the majority of transcribed RNAs consist of noncoding sequences. Among these, long noncoding RNAs (lncRNAs) are a subset characterized by either no or very limited protein-coding capacity, with a length exceeding 200 nucleotides. These transcripts can undergo epigenetic modifications, with the most common being m6A and m5C. In cancers, m5C can alter the expression of downstream genes and influence tumor characteristics by regulating lncRNAs. For various cancer types, including pancreatic cancer, lung cancer, prostate cancer, breast cancer, and bladder cancer, m5C-related lncRNAs can serve as tumor biomarkers to predict the prognosis and efficacy of treatment.^[9]^ However, to date, no research has explored the clinical significance of m5C-related lncRNAs in RCC. In the present study, we extracted the expression data of m5C-regulated genes and lncRNAs from The Cancer Genome Atlas (TCGA) database. Using Pearson correlation analysis, we identified m5C-related lncRNAs, constructed a predictive model to forecast the overall prognosis of RCC patients, and assessed their sensitivity to target and immunotherapy to provide new insights into personalized therapies for RCC.

## 2. Methods

### 2.1. Extraction of patients’ information and selection of m5C-related lncRNAs in RCC

This study was performed based on the data from public database TCGA (https://www.cancer.gov/ccg/research/genome-sequencing/tcga), which did not require ethical approval. We directly downloaded RNA-seq transcriptome data, relevant clinical information, and mutation data of RCC patients from TCGA, encompassing the kidney chromophobe, kidney renal clear cell carcinoma, and kidney renal papillary cell carcinoma databases. To minimize statistical bias, patients with missing survival data were excluded. Based on a literature review, we identified 27 m5C genes and obtained the expression matrix of lncRNA and m5C genes from the RNA-seq transcriptome data of patients with TCGA RCC. Differential box plots (boxplots) and heatmaps for m5C regulatory genes were generated using the R software (version 4.2.1). A protein–protein interaction (PPI) network for m5C genes was constructed using the STRING database (https://cn.string-db.org), with a minimum interaction score requirement of 0.95. Pearson correlation analysis was then employed to calculate the associations among m5C regulatory factors, which were visually represented using the Corrplot R package. Co-expression analysis was conducted using the limma package with Wilcoxon test, and the filtering criteria were set as |cor| > 0.7, *P* < .001. Finally, we identified 441 m5C-related lncRNAs.

### 2.2. Establishment and validation of the risk model

TCGA dataset was randomly divided into training and testing groups. The training set was used to construct the m5C-related lncRNA risk model, whereas the testing set was employed to validate the established model. Table S1, Supplemental Digital Content, https://links.lww.com/MD/P300 presents the baseline characteristics of the 2 groups. A chi-square test was conducted, and a *P* value >.05 indicated no significant differences in clinical features between the 2 groups. After merging the expression data of m5C-related lncRNAs and the survival information of RCC patients from TCGA database, univariate Cox regression analysis was employed to filter out lncRNAs significantly associated with prognosis from the pool of 441 m5C-related lncRNAs (*P* < .001). Using the R package “glment,” least absolute shrinkage and selection operator-COX regression analysis identified 7 lncRNAs (HM13-IT1, AP000331.1, COLCA1, AC130650.1, AC090337.2, AC010285.3, and AC007620.3) significantly correlated with the overall survival (OS). Subsequently, using multivariate Cox regression analysis, we selected 3 lncRNAs (HM13-IT1, COLCA1, and AC010285.3) to establish the risk model. The risk score was calculated as follows: risk score = 0.242349903601839 × expr (HM13-IT1) + (−0.707651063794831) × expr (COLCA1) + 1.01552791772116 × expr (AC010285.3). Here, 0.242349903601839, −0.707651063794831, and 1.01552791772116 are the survival-related coefficients for HM13-IT1, COLCA1, and AC010285.3, respectively and expr (lncRNA) represents the expression level of the respective lncRNA. The samples were classified into high- and low-risk groups based on the median risk score.

### 2.3. Survival analysis and principal component analysis (PCA)

Kaplan–Meier survival analysis was conducted using the R packages “survminer” and “survival” to assess the differences in OS between the high and low-risk groups. Considering additional clinical features, such as pathological pattern, age, sex, and pathological pattern, both univariate and multivariate Cox regression analyses were performed to examine whether the risk score model serves as an independent prognostic variable. Receiver operating characteristic (ROC) curves were used to compare the predictive efficacy of the risk score with that of the other independent risk factors. Subgroup analyses were conducted based on factors such as age, sex, TNM stage, and pathological type. Furthermore, to address high-dimensional data, including the entire gene expression profile, m5C-related genes, m5C-related lncRNAs, and risk model, PCA was applied. PCA was used for dimensionality reduction and visualization, providing insights into the overall structure and relationships within the data.

### 2.4. Nomogram construction

Using the R package “regplot,” a nomogram was created, incorporating patients’ clinical characteristics and risk scores to predict patient survival rates at 1, 3, and 5 years. The calibration curve, based on the Hosmer–Lemeshow goodness-of-fit test, was utilized to demonstrate the consistency between the actual results and model predictions. A c-index curve was used to assess the predictive accuracy of the model.

### 2.5. Identification of risk-associated genes and functional enrichment analysis

We also employed the limma package to identify differentially expressed genes between the high- and low-risk groups. Subsequently, the R package clusterProfiler was utilized for gene ontology (GO) enrichment analysis, where the analysis threshold was determined by both *P*-value and q-value. The significance of functional enrichment was considered when both values were <.05.

### 2.6. Mutation burden, immune function, and immune evasion prediction

We assessed and aggregated the mutation data using the R package “maftools.” Tumor mutation burden (TMB) was measured based on tumor-specific mutated genes. Employing the R package “GSVA,” we conducted single-sample gene set enrichment analysis to explore differences in immune functions between the high- and low-risk groups. Additionally, the tumor immune dysfunction and exclusion (TIDE) algorithm was used to predict immune treatment responses in the high- and low-risk groups.

### 2.7. Prediction of targeted drug efficacy

The R package “oncoPredict” was used to assess whether the risk score model could be employed to predict the sensitivity of targeted drugs for RCC. The effectiveness of various targeted drugs in renal cancer cells was represented by the half-maximal inhibitory concentration (IC50) of the respective drugs. The drug response data were obtained from https://osf.io/c6tfx/.

## 3. Results

### 3.1. The expression of m5C enzymes are dysregulated in RCC

**Figure 1.**
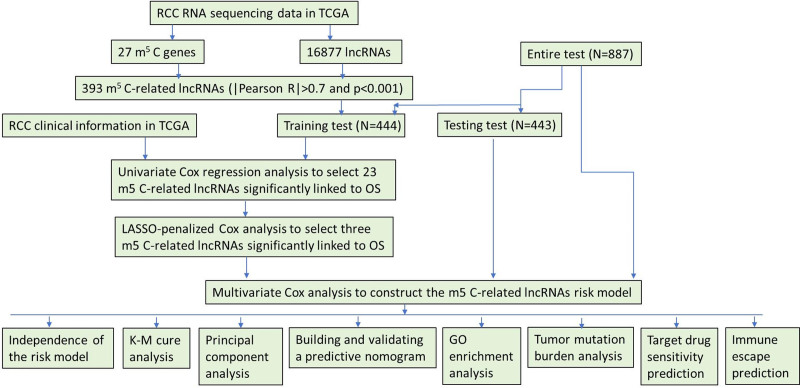
Flow chart of this study.

**Figure 2.**
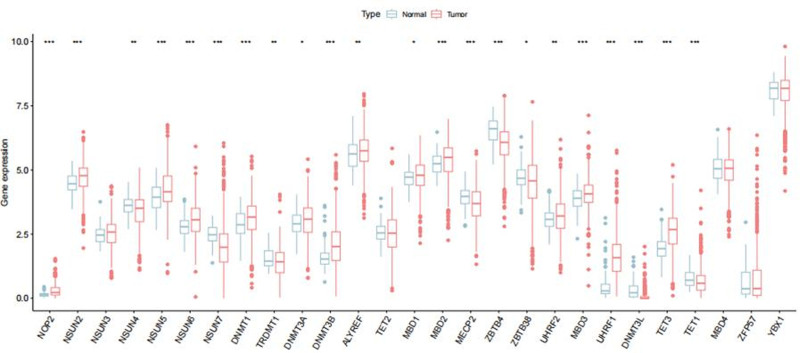
Box-plots of the gene expression of m5C regulating enzymes in RCC (Tumor) and normal tissues (Normal). When *P* < .001, it is shown as ***, *P* < .01 as **, *P* < .05 as *. m5C = 5-methylcytosine, RCC = renal cell carcinoma.

**Figure 3.**
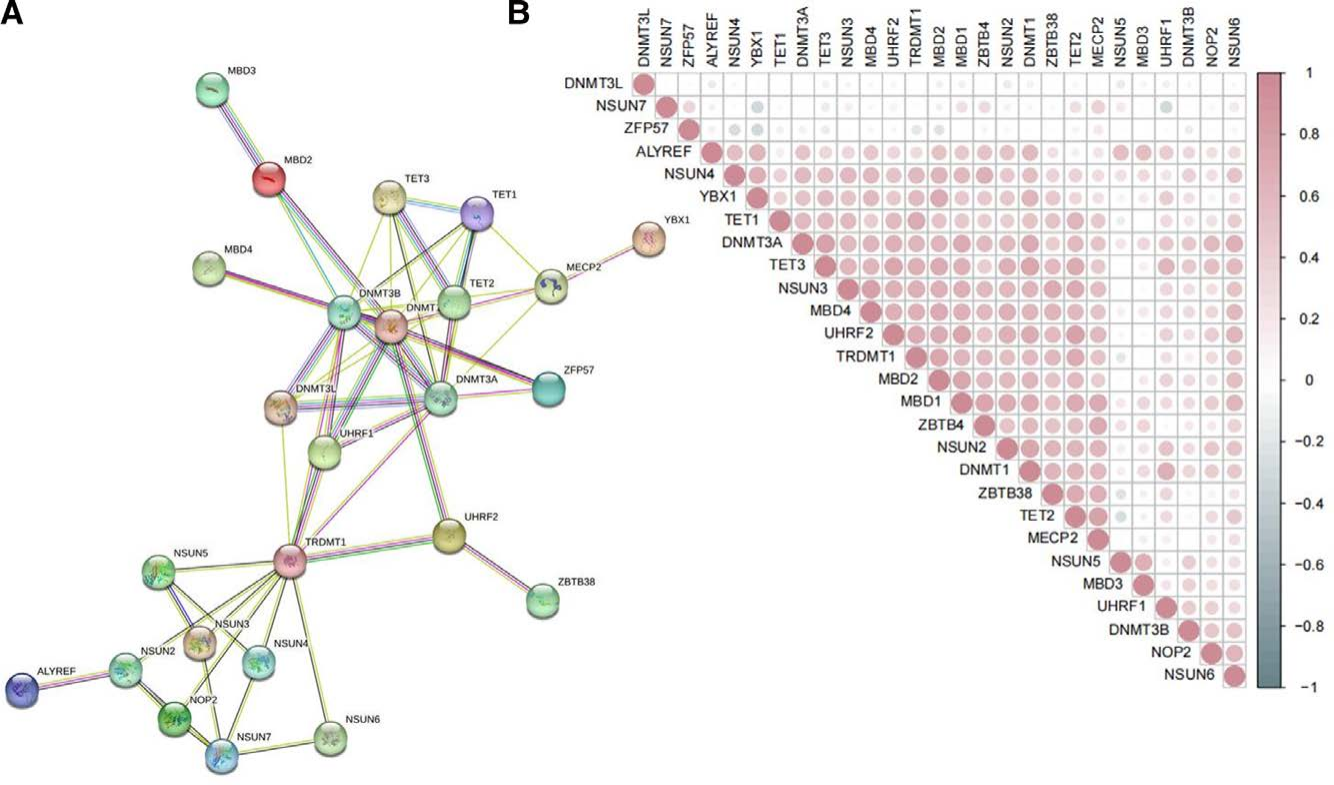
(A) The protein–protein interaction (PPI) network among m5C regulating enzymes (B) Pearson correlations between m5C regulating enzymes, red means positive correlation, blue means negative correlation. m5C = 5-methylcytosine.

A flowchart of the study is shown in Figure 1. We observed variations in the expression of m5C regulatory factors between RCC and normal renal tissues. RNA-seq data, comprising 129 normal adjacent tissues and 899 RCC tissues, were retrieved from TCGA database. Heatmaps and vioplots were generated based on the data to illustrate the expression patterns of m5C-related genes in RCC (Fig. 2 and Figure S1, Supplemental Digital Content, https://links.lww.com/MD/P301). The results revealed a significant upregulation (*P* < .05) in the expression levels of NOP2, NSUN2, NSUN5, NSUN5, DNMT1, DNMT3A, DNMT3B, ALYREF, MBD1, MBD2, MBD3, UHRF1, UHRF2, and TET3 in the tumor tissues. Conversely, the expression of NSUN7, MECP2, ZBTB4, ZBTB38, DNMT3L, and TET1 was significantly downregulated (*P* < .05). Subsequently, a PPI network was constructed based on the STRING database (Fig. 3A), revealing DNMT3A, DNMT1, TET1, TET2, MECP2, and TRDMT1 as the 6 strongest interconnected PPI nodes. This correlation was further validated by co-expression analysis and quantified using Pearson correlation coefficient (Fig. 3B). These findings highlight significant internal correlations among m5C regulatory genes in RCC.

### 3.2. Identification of m5C-associated lncRNAs and establishment of a prognostic model

**Figure 4.**
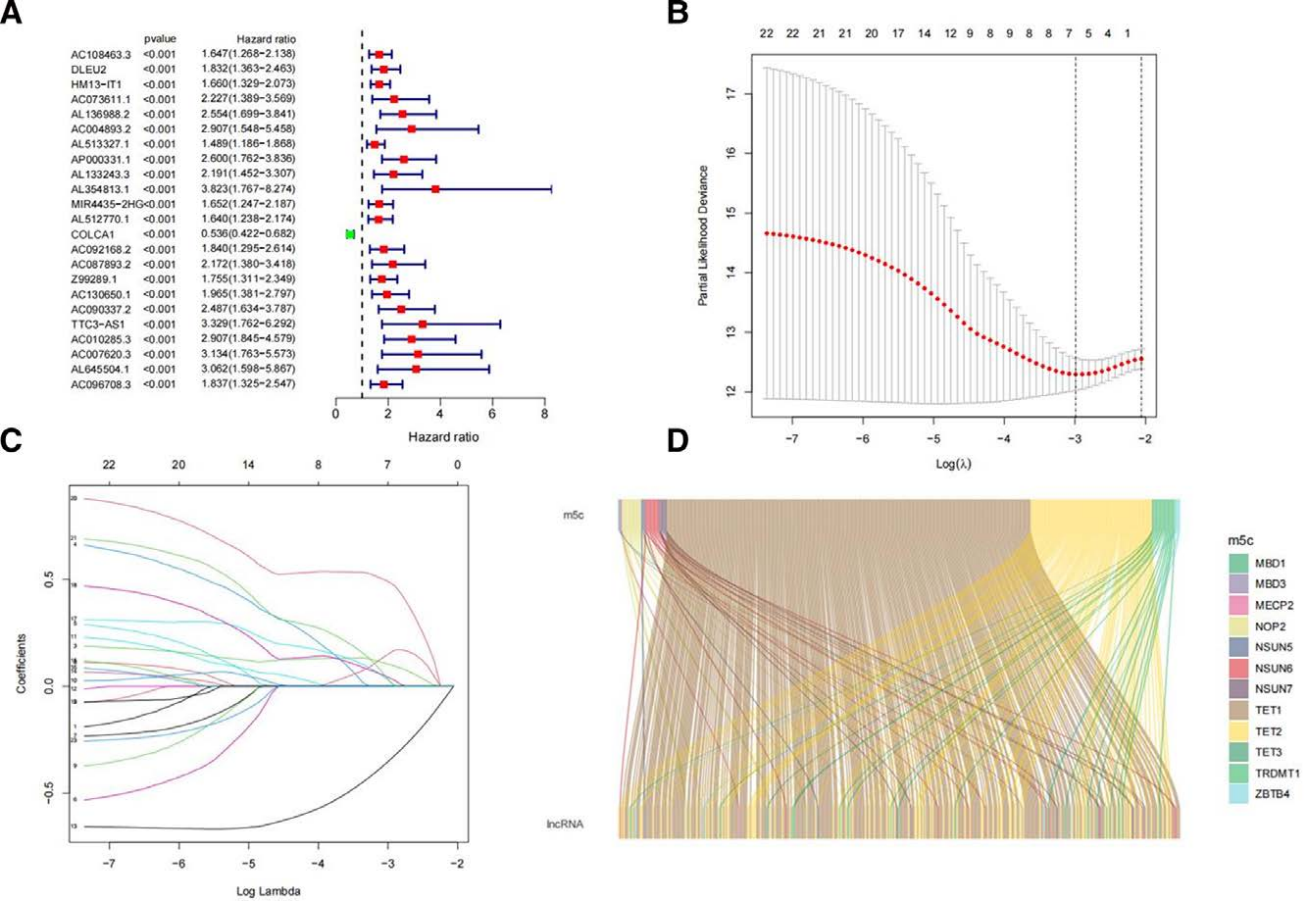
Identification of prognosis-associated lncRNAs and establishment of a prognostic model (A) Forest plot of 23 prognosis-associated lncRNAs. Risk factors are shown in red, and protective factors are shown in green. (B) The tuning parameters (log l) of OS-related proteins were selected to cross-verify the error curve. According to the minimal criterion and 1-se criterion, perpendicular imaginary lines were drawn at the optimal value. (C) The LASSO coefficient profile of prognosis-related lncRNAs and perpendicular imaginary line were drawn at the value chosen by 10-fold cross-validation. (D) Sankey relational diagram for m5C genes and m5C-related lncRNAs. LASSO = least absolute shrinkage and selection operator, lncRNAs = long noncoding RNAs, m5C = 5-methylcytosine, OS = overall survival.

**Figure 5.**
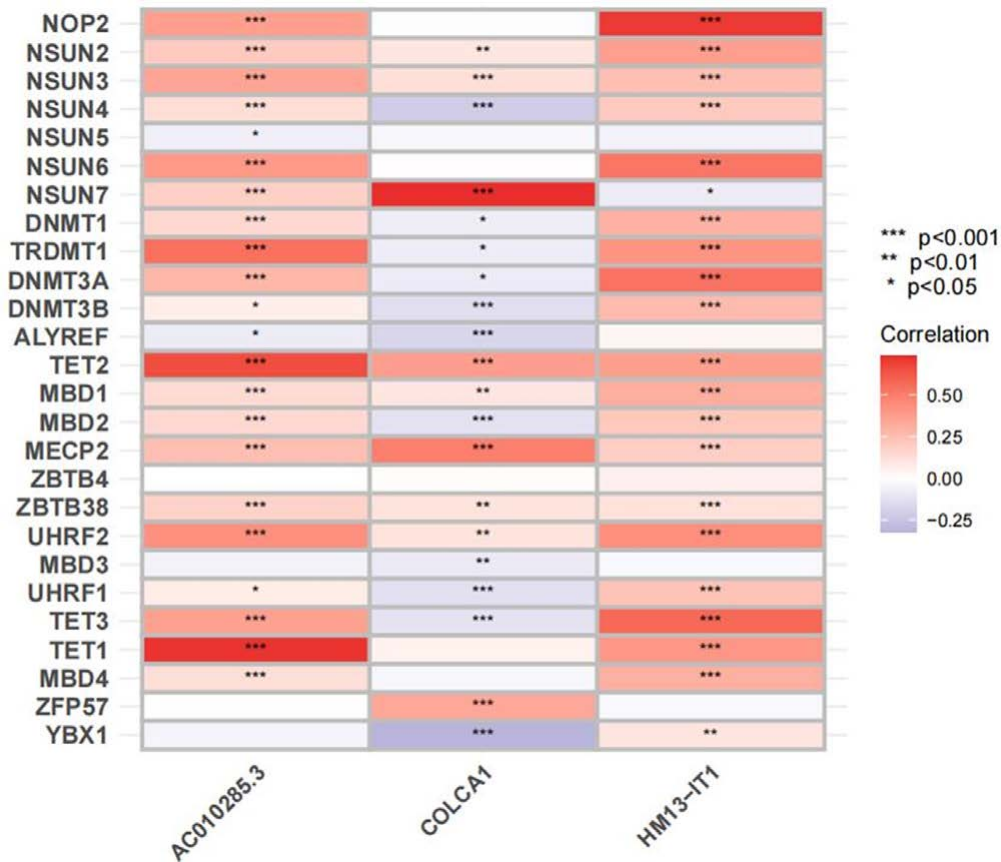
Heatmap for the correlations between m5C genes and the 3 prognostic m5C-related lncRNAs. lncRNAs = long noncoding RNAs, m5C = 5-methylcytosine.

**Figure 6.**
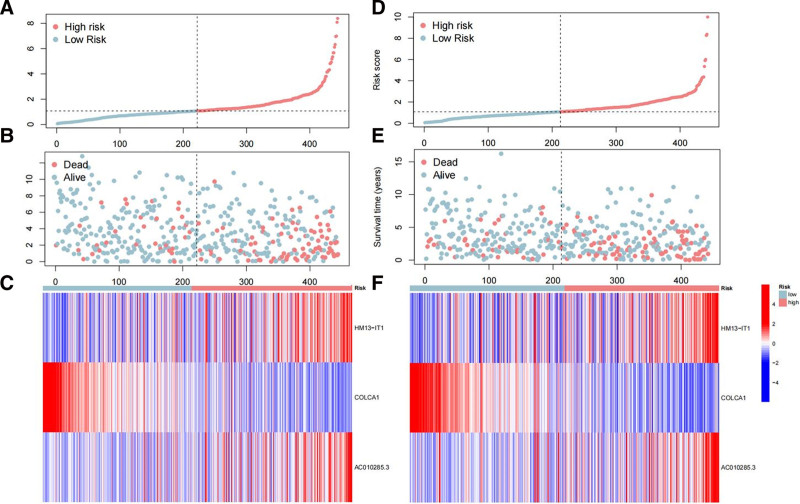
(A) Distribution of m5C-related lncRNA model-based risk score in the training set. (B) Different patterns of survival status and survival time between the high- and low-risk groups in the training set. (C) Clustering analysis heatmap shows the expression standards of the 3 prognostic lncRNAs for each patient in the training set. (D) Distribution of m5C-related lncRNA model-based risk score in the testing set. (E) Different patterns of survival status and survival time between the high- and low-risk groups in the testing set. (F) Clustering analysis heatmap shows the expression standards of the 3 prognostic lncRNAs for each patient in the testing set. lncRNAs = long noncoding RNAs, m5C = 5-methylcytosine.

In this study, m5C-associated lncRNAs were defined as those that were significantly correlated with one or more m5C regulatory factors (|correlation coefficient| > 0.6, *P* < .001). As depicted in Figure 4A, we conducted univariate COX regression analysis, identifying 23 m5C-associated lncRNAs that were significantly associated with the prognosis of patients with RCC. Least absolute shrinkage and selection operator Cox regression analysis led to the construction of a prognostic model comprising 3 m5C-associated lncRNAs, represented by the following formula: Risk score = 0.242349903601839 × expr (HM13-IT1) − 0.707651063794831 × expr (COLCA1) + 1.01552791772116 × expr(AC010285.3) (Fig. 4B, C). Using this formula, the median risk score in the training group was calculated as 1.0770565, serving as the cutoff point for distinguishing high- and low-risk groups in both the training and test sets. A Sankey plot was used to visualize the co-expression network of m5C-associated lncRNAs, illustrating the relationship between m5C regulatory factors and m5C-associated lncRNAs (Fig. 4D). Subsequently, correlation heatmaps (Fig. 5) were generated to show the relationships between the 3 lncRNAs used to construct the risk model and m5C regulatory factors. To assess the predictive capability of the risk model, a unified risk-score calculation formula was applied to compute the risk scores for each patient in both the training and test sets (Fig. 6). Figure 6A–C and D–E describe the distribution of risk levels, survival status patterns, survival time, and expression of m5C-associated lncRNAs in the training and test sets, respectively. Patients with higher risk scores experienced more death events, with prognostically adverse lncRNAs significantly overexpressed in the high-risk group and protective lncRNAs significantly overexpressed in the low-risk group. The distribution of risk levels, survival status patterns, survival time, and expression of m5C-associated lncRNAs for the entire dataset are presented in (Figure S2, Supplemental Digital Content, https://links.lww.com/MD/P301).

### 3.3. Evaluation of the prognostic value of the risk model

**Figure 7.**
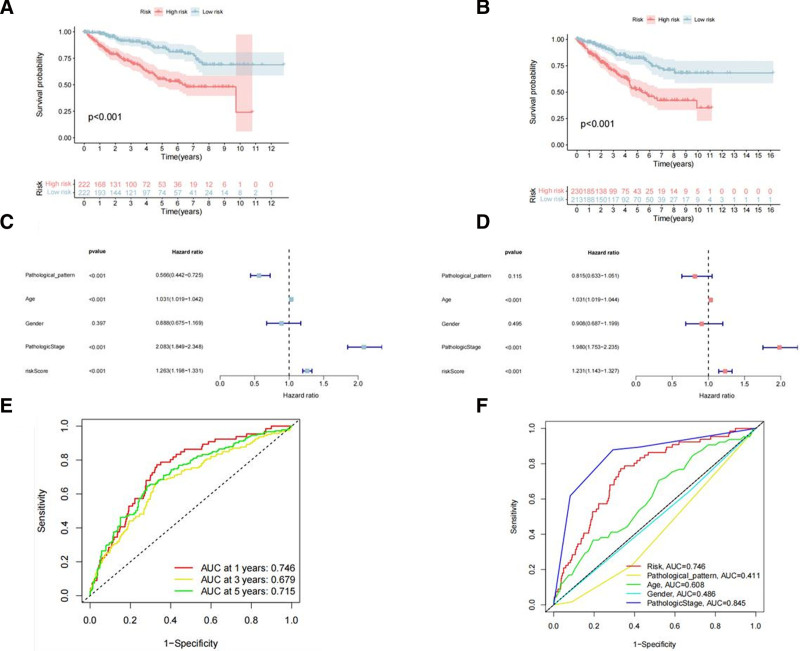
(A) The Kaplan–Meier curve for OS between high and low-risk groups in training set. (B) The Kaplan–Meier curve for OS between high and low-risk groups in training set in testing set. (C, D) Univariate and multivariate analyses of the clinical characteristics and risk score with the OS. (E) ROC curves of risk score for 1year, 3 year and 5 year OS. (F) ROC curves of the clinical characteristics and risk score for 3 year OS. OS = overall survival, ROC = receiver operating characteristic.

**Figure 8.**
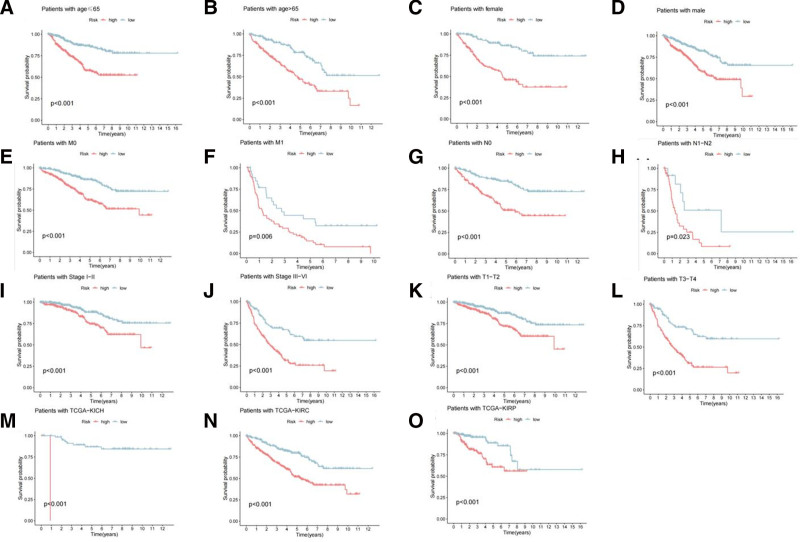
Kaplan–Meier curves of OS differences stratified by: (A, B) age, (C, D) gender, (E, F) M stage, (G, H) N stage, (I, J) (K, L) T stage, (M, N, and O) pathological type between the high- and low-risk groups. OS = overall survival.

The results of Kaplan–Meier survival analysis conducted on both the training and test sets consistently indicated that patients with higher risk scores in RCC had shorter OS than those with lower risk scores (Fig. 7A, B). The Kaplan–Meier survival analysis for the entire cohort is shown in (Figure S2, Supplemental Digital Content, https://links.lww.com/MD/P301). Univariate and multivariate Cox regression analyses were performed on the entire cohort to investigate whether the risk-score model was an independent risk factor. The results revealed that an increase in the risk score (95% CI HR: 1.143–1.372; HR = 1.231; *P* < .001), age (95% CI: 1.019–1.044; HR = 1.031; *P* < .001), and pathological stage (95% CI: 1.753–2.235; HR = 1.980; *P* < .001) were all independent risk factors associated with the progression of RCC (Fig. 7C, D). Time-dependent ROC analysis demonstrated that the area under the curve values for the risk model at 1, 3, and 5 years were 0.746, 0.679, and 0.715, respectively (Fig. 7E). Multifactorial ROC analysis revealed that the area under the curve for the 3-year survival rate was 0.746, second only to the pathological stage (Fig. 7F). Additionally, the proposed risk model was further validated in different subgroups divided by sex, age, pathologic stage, and pathological classification. The low-risk group consistently exhibited superior OS compared with the high-risk group (Fig. 8).

### 3.4. PCA further validates the grouping capability of the risk model

**Figure 9.**
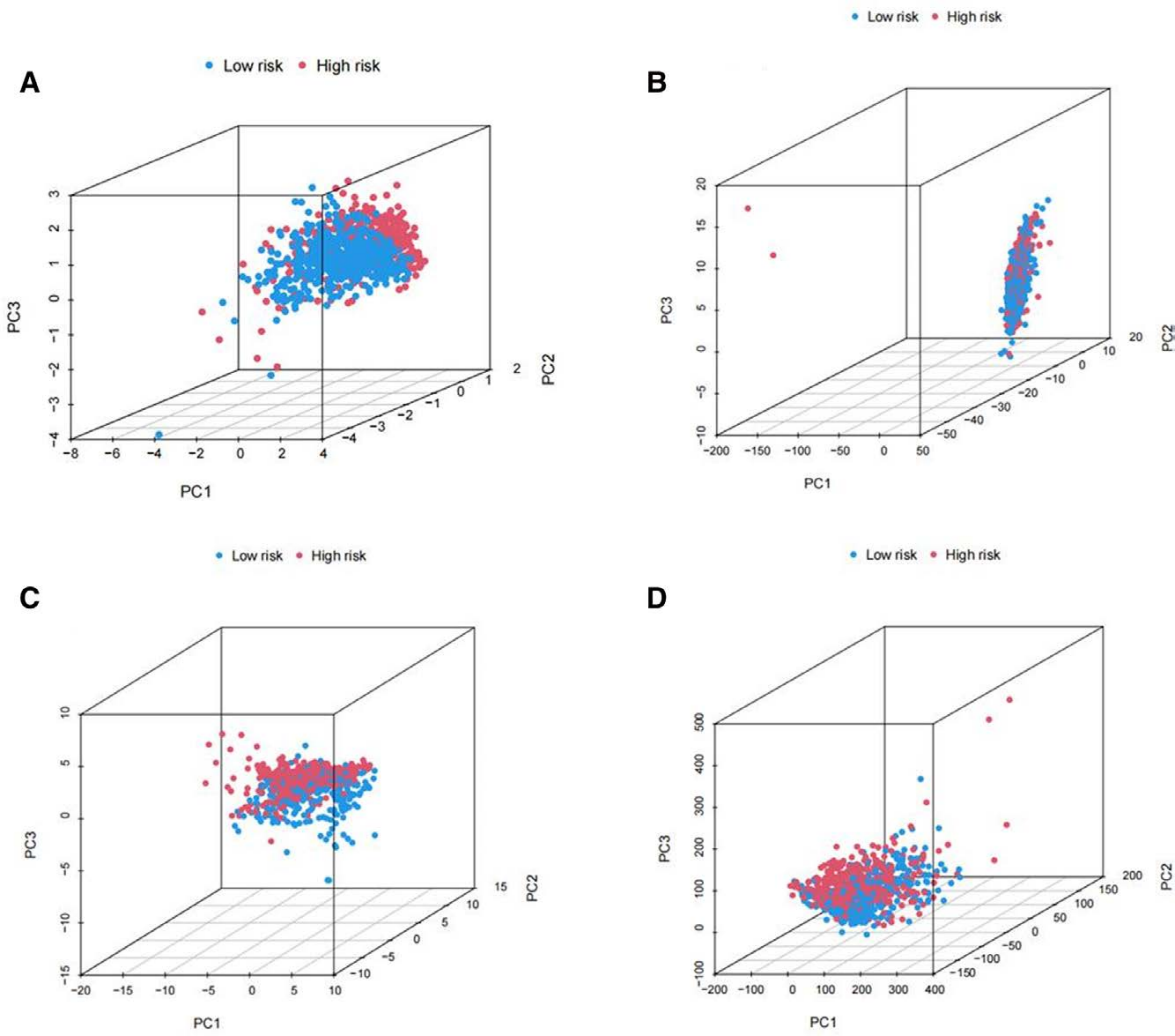
Principal component analysis between the high- and low-risk groups based on (A) entire gene expression profiles, (B) m5C-related lncRNAs, (C) m5C genes, and (D) risk model based on the representation profiles of the 3 m5C-related lncRNAs. lncRNAs = long noncoding RNAs, m5C = 5-methylcytosine.

PCA analysis was conducted to assess the differences between the low-risk and high-risk groups based on the entire gene expression profile, 27 m5C genes, and all m5C-related lncRNAs, and the expression profiles of the 3 lncRNAs were used to establish the risk model. The distribution of the high- and low-risk groups appears to be relatively dispersed (Fig. 9B–D). However, the results obtained from our model indicated distinct distributions between the low- and high-risk groups (Fig. 9A). These findings suggest that the risk model can effectively differentiate between the low- and high-risk groups.

### 3.5. Construction and validation of the nomogram line chart

**Figure 10.**
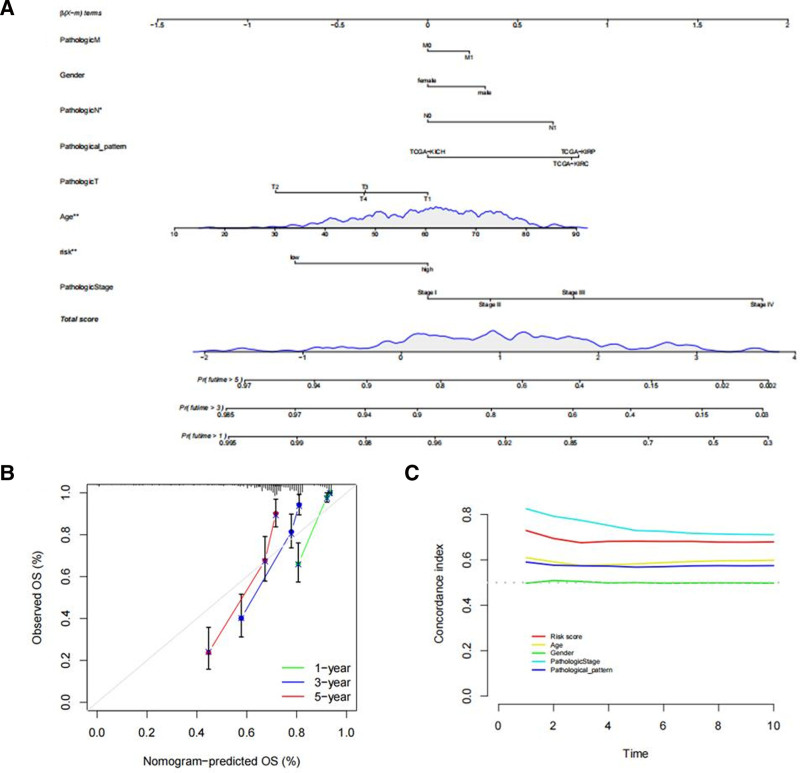
Construction and evaluation of a prognostic nomogram (A) The nomogram predicts the probability of the 1-, 2-, and 3-year OS. (B) The calibration plot of the nomogram predicts the probability of the 1-, 2-, and 3-year OS. (C) The C-index curve of clinical characteristics and risk score. OS = overall survival,

A nomogram was constructed encompassing the risk grade and clinical risk features to predict the occurrence rates of 1-, 3-, and 5-year OS (Fig. 10A). C-index and calibration curves were plotted to better assess the uniqueness and sensitivity of the nomogram. Over an extended duration, the C-index for the risk score consistently maintained a high level, suggesting that the risk scores can feasibly predict the prognosis of RCC (Fig. 10C). Calibration curves for 1, 3, and 5 years further indicated the accurate prediction of patient prognosis by the nomogram (Fig. 10B).

### 3.6. Potential functional pathways of differentially expressed genes between high and low-risk groups

**Figure 11.**
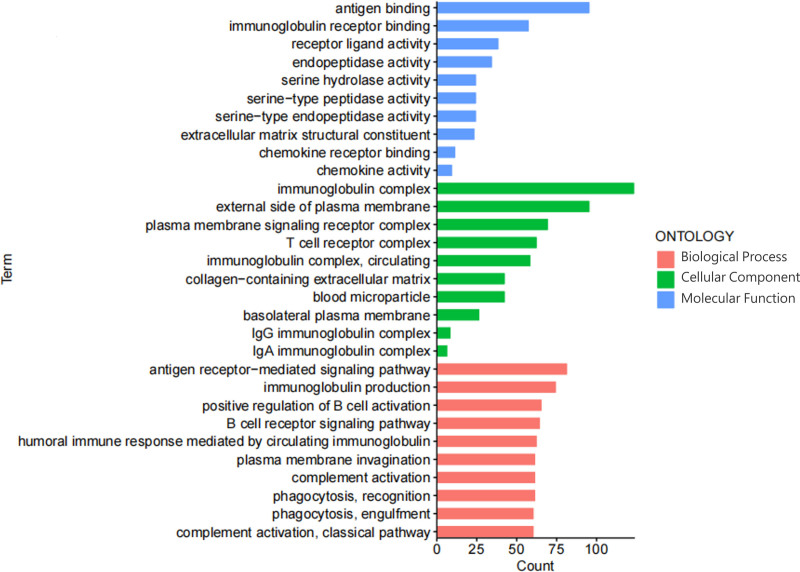
The functional gene ontology (GO) enrichment analysis for the differential expressed genes between high and low-risk groups.

To comprehend the underlying molecular mechanisms between high- and low-risk groups, we conducted analysis using the “limma” R package, with filtering conditions set at logFC > 1 and fdr < 0.05. Next, GO analysis was performed for the filtered set of differentially expressed genes, GO analysis was performed. We observed significant enrichment of differentially expressed genes in pathways related to antigen binding, immunoglobulin complexes, and antigen receptor-mediated signaling pathways (Fig. 11).

### 3.7. TMB based on high and low-risk groups

**Figure 12.**
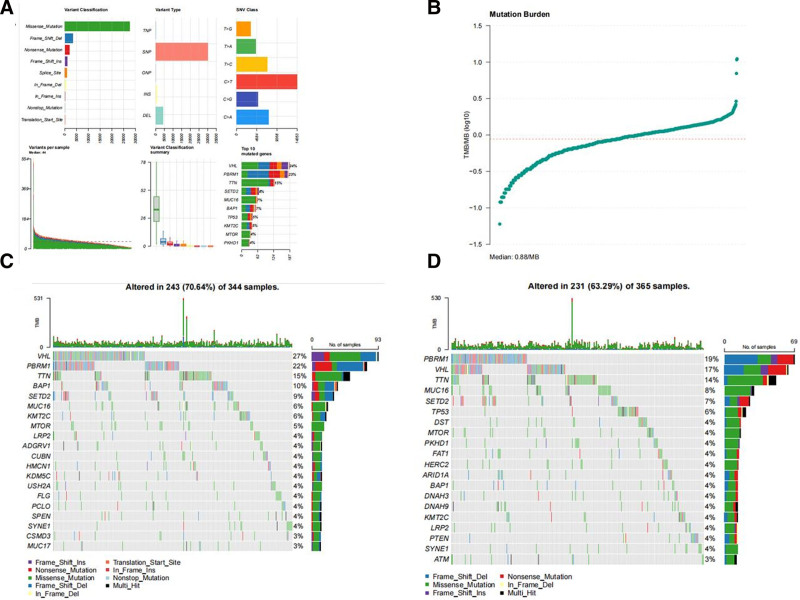
(A) The various types of mutation. (B) The distribution of TMB. Waterfall plot displays mutation information of the genes with high mutation frequencies in the high-risk group (D) and low-risk group (E). TMB = tumor mutation burden.

**Figure 13.**
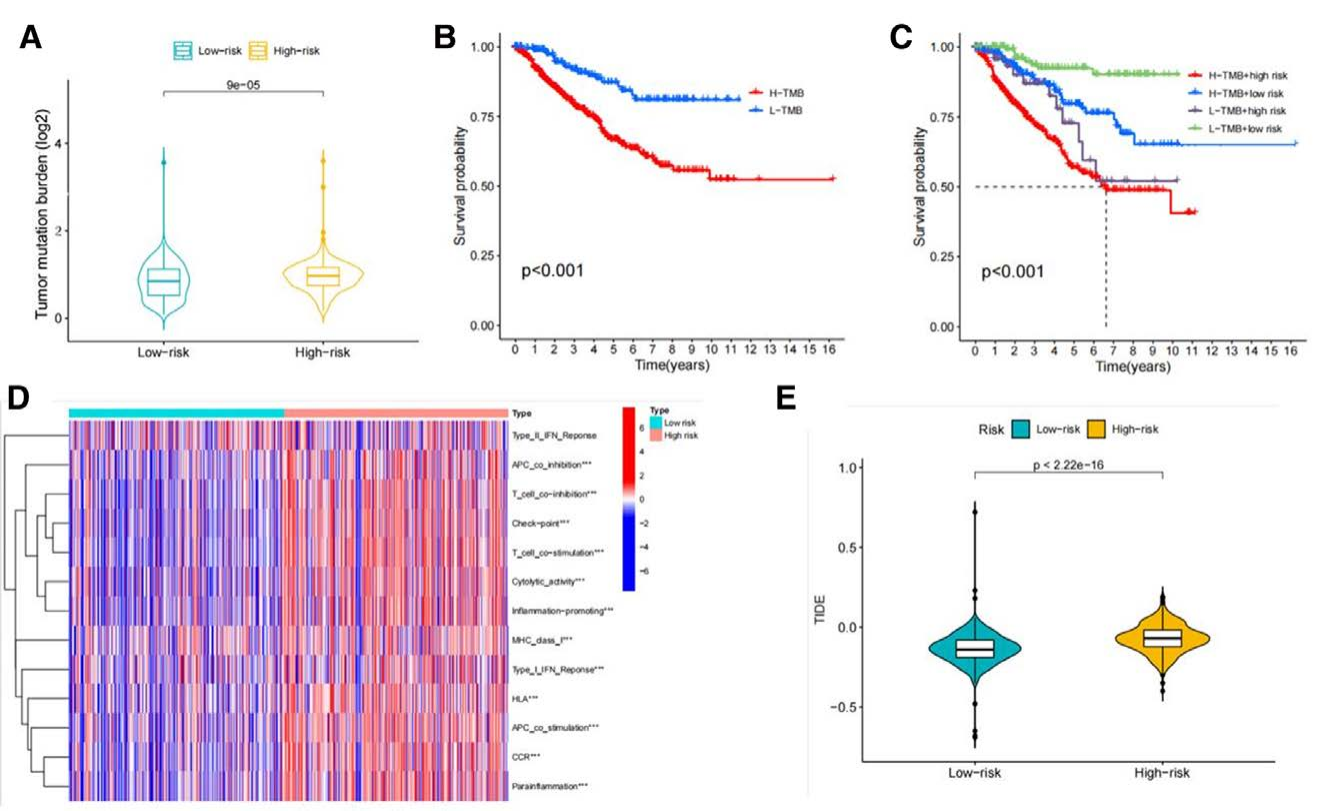
(A) TMB difference between high and low-risk patients. (B) Kaplan–Meier curve of OS between patients with high TMB (H-TMB) and low TMB (L-TMB). (C) Kaplan–Meier curve of OS is shown for patients classified according to the TMB level and m5A-related lncRNA model. (D) The indicated standards of the immunity index for each patient. (E) TIDE prediction difference in the high- and low-risk patients. lncRNAs = long noncoding RNAs, OS = overall survival, TIDE = tumor immune dysfunction and exclusion, TMB = tumor mutation burden.

We used the R package maftools to analyze and summarize the mutation data (Fig. 12A, B). Mutations were stratified based on variant effect prediction factors (Fig. 12C, D), highlighting the top 20 driver genes with the highest alteration frequencies in the high- and low-risk groups. Subsequently, we calculated the TMB score using the TCGA somatic mutation data. TMB in the low-risk group was lower than that in the high-risk group, indicating a high correlation between risk model-based stratification and TMB (Fig. 13A). Concurrently, Kaplan–Meier analysis demonstrated that RCC patients with a high TMB had worse OS than those with a low TMB. Furthermore, when patients exhibited both a high TMB and a high-risk score simultaneously, they experienced the poorest OS prognosis (Fig. 13B, C).

### 3.8. Estimation of tumor immune microenvironment and immunotherapeutic response using the risk model

Building upon the m5C-related lncRNA risk model established earlier, we conducted further analysis of the enrichment levels and activities of several immune cells, pathways, or functions in RCC. The expression differences in immune indicators between the low- and high-risk groups were statistically significant (Fig. 13D). These results are consistent with our previous findings from the GO enrichment analysis, which revealed close associations with immune functions. Subsequently, we investigated the correlation between the prognostic model and the immunotherapy. As expected, the high-risk group had higher TIDE scores than the low-risk group, indicating a poorer response to immunotherapy. This suggests that the risk model can predict the effectiveness of immunotherapy for RCC (Fig. 13E).

### 3.9. Relationship between risk score and sensitivity to targeted therapy

**Figure 14.**
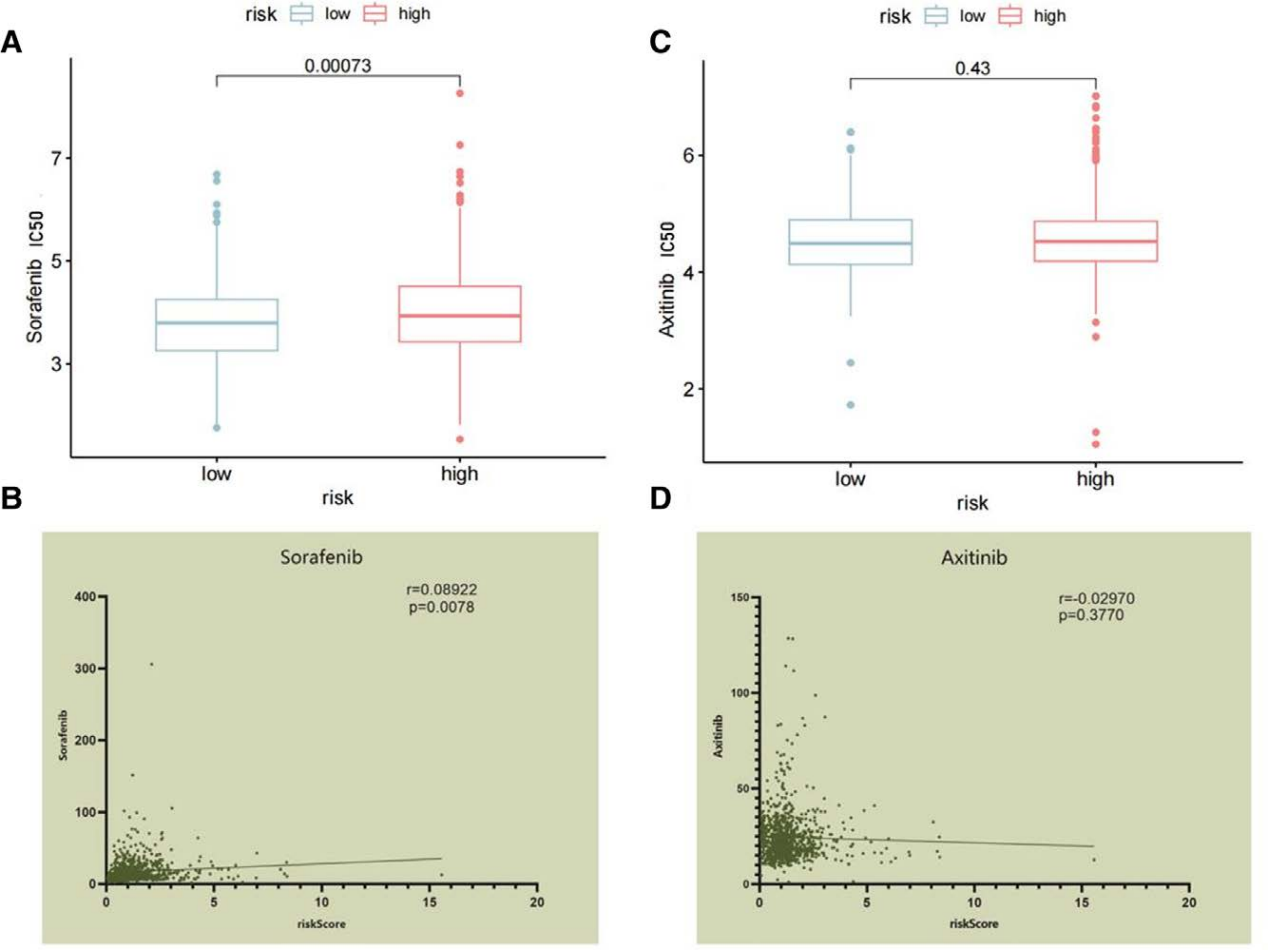
The predicted sensitivity to target drugs. (A) The different IC50 values for sorafenib in high and low-risk groups. (B) The correlation between risk score and IC50 of sorafenib. (C) The different IC50 values for axitinib in high and low-risk groups. (D) The correlation between risk score and IC50 of axitinib.

We utilized the R package “oncoPredict” to explore the sensitivity differences in drug treatment between the high-risk and low-risk groups, creating IC50 boxplots and correlation scatter plots (Fig. 14A–D). We observed that patients in the high-risk group had higher IC50 values for sorafenib treatment and there was a positive correlation with the risk score. This indicated that patients with higher risk scores were less sensitive to sorafenib (*P* < .05). However, axitinib treatment did not show statistical significance (*P* > .05).

## 4. Discussion

In recent years, researchers have been investigating novel treatment methods for RCC and have made significant progress. Particularly for metastatic renal cancer, the risk stratification of patients has become a crucial assessment tool for determining prognosis.^[10]^ Simultaneously, with an in-depth understanding of the molecular mechanisms of RCC, molecular regulation has become a vital marker for determining patient prognosis and has been incorporated into the latest WHO pathological classification standard.^[11]^ Targeted therapy and immunotherapy are currently the preferred systemic treatments for metastatic RCC. However, there is considerable variability in treatment outcomes, primarily due to differences in molecular characteristics, including gene methylation, leading to variations in tumor characteristics and drug sensitivity.^[12,13]^ Personalized specific treatment is the direction to enhance treatment effectiveness and patient prognosis. Therefore, it is crucial to identify reliable and stable molecular markers based on RCC molecular characteristics.

Noncoding RNAs have been found to play a key role in the regulation of various diseases, particularly in the occurrence and development of cancers. LncRNAs have been confirmed to act as diagnostic and therapeutic markers in various kidney diseases, including acute kidney injury, chronic kidney disease, and RCC.^[14]^ They interact with various m5C-regulating proteins. For instance, NSUN2 can bind and methylate nucleotide metabolism regulator lncRNAs with m5C modification, promoting drug resistance in esophageal cancer.^[15]^ NSUN2 enhances the stability of lncRNA H19, thereby promoting the deterioration of liver cell carcinoma.^[16]^ The M5C readers, ALYREF and YBX1, can regulate the expression and degradation of lncRNAs, promoting tumor progression.^[17,18]^ The M5C eraser TET is generally considered to regulate DNA demethylation, but recent studies suggest that it can also regulate the expression and degradation of lncRNA.^[19]^

In this study, a predictive model for RCC was constructed based on m5C-related lncRNAs including HM13-IT1, COLCA1, and AC010285.3. Among them, COLCA1 is considered to be involved in oxidative stress responses and serves as a biomarker for various malignant diseases such as lung cancer, gastric cancer, and colon cancer.^[20,21]^ In RCC, COLCA1 has been identified as a biomarker for predicting early recurrence after nephrectomy and OS.^[22,23]^ HM13-IT1 functions as an m6A-related lncRNA and acts as a biomarker for endometrial cancer.^[24]^ There is no literature on AC010285.3 in cancer. The total survival data of patients could be accurately predicted using our predictive model based on these 3 lncRNAs. The high-risk group is associated with a shorter OS time and is more precise than age, sex, and pathological grading. Consistent results were observed in subgroup analyses, considering different age, sex, TNM stage, and pathological type. Furthermore, the nomogram validated the predictive model.

Immune checkpoint inhibitors (ICI) have gradually become the first-line choice for the systemic treatment of various malignancies, such as melanoma, non-small cell lung cancer, and urothelial carcinoma. In these tumors, high TMB often signifies a better response to ICI.^[25]^ Conversely, for some tumors with low TMB, such as prostate cancer and pancreatic cancer, responsiveness to ICI is generally poor. However, RCC presents a unique scenario; despite having a lower TMB, it exhibits a relatively favorable response to ICI. A possible reason for this phenomenon is that RCC, within its limited mutations, specifically generates a substantial number of neoantigenic peptides.^[26]^ Therefore, while TMB serves as a marker for the efficacy of ICI and posttreatment prognosis in most tumors, its predictive value in RCC still requires further research and validation. The TIDE score has been widely recognized as a predictive measure of tumor responsiveness to ICI.^[27]^ In this study, the high-risk group was found to have high TMB and high TIDE. This suggests that not only does the high-risk group have a poor prognosis, but it also exhibits diminished effectiveness in response to ICI.

This model can also predict the responsiveness to targeted therapies. The results indicated that the high-risk group showed reduced responsiveness to sorafenib, whereas there was no significant difference in the treatment efficacy of axitinib between the high-risk and low-risk groups. Previous studies have suggested that m5C regulates tumor drug resistance through various mechanisms, but no consensus has been reached.^[28]^ In this study, only the efficacy data for sorafenib and axitinib were obtained, and the sample size was limited, preventing us from reaching definitive conclusions.

We have to admit that there are inherent limitations in our study. Firstly, functional assays based on tissue or cell samples should be performed to confirm the biological relevance of the identified lncRNAs, which may be our next research program. Secondly, our study failed to provide more validated conclusion because of the retrospective nature and the heterogeneities among the included data.

In conclusion, this study established a novel predictive model for RCC based on m5C-related lncRNAs, enabling a relatively accurate prediction of patient OS. Through our analysis, we identified the potential to predict patient responses to both immunotherapy and targeted therapy. This offers a new tool for enhancing clinical services and providing personalized treatment plans. However, it is important to note that this study relied solely on the analysis of existing database data, and its conclusions require validation through clinical practice.

## Acknowledgments

We acknowledge the members and all participants of The Cancer Genome Atlas.

## Author contributions

**Investigation:** Jin Wu.

**Methodology:** Baosai Lu, Chenming Zhao.

**Software:** Jin Wu, Yalin Niu.

**Validation:** Yuewei Yin.

**Writing – original draft:** Chenming Zhao.

**Writing – review & editing:** Chenming Zhao.

## Supplementary Material


