# Supplementary material for: Construction of a risk model based on m5C-associated lncRNAs to predict the prognosis in renal cell carcinoma
Source: Medicine (Baltimore). 2025 Jul 4;104(27):e43052. doi: 10.1097/MD.0000000000043052 (PMC12237355; doi:10.1097/MD.0000000000043052)
Supplement: Supplementary file 1 [file medi-104-e43052-s001.docx]

**Table 1** Summary of clinicopathological parameters of the study cohorts.

|  | **Training Cohort**  **Number（%）** | **Testing Cohort**  **Number（%）** | ***p* value** |
| --- | --- | --- | --- |
| **Pathological pattern** |  |  | 0.1598 |
| KICH | 31(7%) | 34(7.66%) |  |
| KIRC | 280(63.21%) | 253(56.98%) |  |
| KIRP | 132(29.8%) | 157(35.36%) |  |
| **Gender** |  |  | 0.685 |
| Female | 142(32.05%) | 149(33.56%) |  |
| Male | 301(67.95%) | 295(66.44%) |  |
| **Age** |  |  | 0.796 |
| ≤60 | 292(65.91%) | 288(64.86%) |  |
| ＞60 | 150(33.86%) | 155(34.91%) |  |
| unknown | 1(0.23%) | 1(0.23%) |  |
| **Pathological Stage** |  |  | 0.8456 |
| Stage I | 221(49.89%) | 237(53.38%) |  |
| Stage II | 52(11.74%) | 51(11.49%) |  |
| Stage III | 100(22.57%) | 89(20.05%) |  |
| Stage IV | 54(12.19%) | 50(11.26%) |  |
| unkown | 16(3.61%) | 17(3.83%) |  |

KICH = Kidney Chromophobe Cell Carcinoma, KIRC = Kidney Renal Clear Cell Carcinoma, KIRP = Kidney Renal Papillary Cell Carcinoma
