# Supplementary material for: Construction of a risk model based on m5C-associated lncRNAs to predict the prognosis in renal cell carcinoma
Source: Medicine (Baltimore). 2025 Jul 4;104(27):e43052. doi: 10.1097/MD.0000000000043052 (PMC12237355; doi:10.1097/MD.0000000000043052)
Supplement: Supplementary file 2 [file medi-104-e43052-s002.docx]

Supplement Figure 1: Heatmap for m5C regulating enzymes in RCC (T) and normal tissues (N). Blue means low level of expression; Red means high level of expression.

Supplement Figure 2: (A) Distribution of m5C-related lncRNA model-based risk score in the entire set. (B) Different patterns of survival status and survival time between the high- and low-risk groups in the entire set. (C) Clustering analysis heatmap shows the expression standards of the three prognostic lncRNAs for each patient in the entire set. (D) The Kaplan-Meier curve for the OS in the entire set.
